# Obstructive Sleep Apnea: New Perspective

**DOI:** 10.3390/medicina59010075

**Published:** 2022-12-29

**Authors:** Salim Surani, Pahnwat Taweesedt

**Affiliations:** 1 Department of Pulmonary, Critical Care & Sleep Medicine, Texas A&M University, College Station, TX 77843, USA; 2 Division of Sleep Medicine, Department of Psychiatry and Behavioral Sciences, Stanford University School of Medicine, Palo Alto, CA 94063, USA

Obstructive sleep apnea (OSA) is one of the most common sleep disorders globally. OSA affects 936 million adults aged 30–69. Obesity, aging, gender, large neck size, craniofacial abnormalities, alcohol, and menopause have been described as risk factors for OSA. In the United States of America (USA), the “obesity capital of the world,” the prevalence of OSA is 33.9% among men and 17.4% among women, as determined by the criteria of apnea–hypopnea index (AHI) >5 (1). When the requirement of AHI >15 is used, the prevalence of OSA is 13% and 6% among men and women, respectively [1]. Surprisingly, China, where the prevalence of obesity is low, also has a high prevalence of OSA, with 24.2% of males and females affected (with no gender difference), suggesting craniofacial and other phenotypes abnormalities may be causing this high prevalence rate [2]. Additional countries provide evidence that there is a multitude of contributing factors to consider when taking into account OSA prevalence, as Germany, Japan, and Poland have a prevalence of OSA exceeding 50% [1,3]; it is necessary to understand these varying factors in order to handle global health issues pertaining to sleep-related breathing disorders [1,3].

Sleep apnea has been shown to cause sleep fragmentation and sleep deprivation, which can cause obesity, cardiovascular complications, poor work performance, occupational hazards, and increased motor vehicle accident risks. Appropriate sleep quality and quantity are an integral part of good health. Kania et al. discuss how features, e.g., female biological sex and chronic heart failure, are independent risk factors for poor sleep quality in patients with OSA [4]. 

The airline and transportation industries have formulated robust screening and compliance therapy policies, whereas the healthcare industry lags [5]. The safety concerns associated with the OSA have been well established, though diagnosis and compliance with therapy remain a concern. Initially, screening tools such as the Epworth Sleepiness Scale, Berlin questionnaire, NAMES criteria, and STOP-Bang criteria have been utilized. Lately, research and emphasis have been placed on artificial intelligence, which can help with screening and diagnosing OSA before patients present with symptoms [6]. Continuous Positive Airway Pressure therapy (CPAP) has been the gold standard for therapy, but alternate surgical and non-surgical therapies are emerging. Even though CPAP therapies are highly effective, compliance and patient acceptance remain low. Using the adherence definition of more than 4 h of usage per night, the non-adherence rate in patients with OSA ranges from 46 to 83% [7].

More data are emerging regarding using patient-centered therapy for OSA, considering various etiologies and features that affect patients and may facilitate the treatment plan. This special edition helps to explore diverse topics related to OSA, including the issues of the importance of clinical and polysomnographic features, the pharmacological aspects of the treatment of OSA, cardiovascular complications of OSA, OSA in patients who have implantable cardiac devices, and the polysomnographic indices following upper airway surgeries. In addition, the diagnosis of OSA through methods of nocturnal polysomnographic using AI and patient-centered therapy for OSA will be discussed.

The emphasis over the years to utilize CPAP therapy and minimize other alternative and surgical therapies has led to an increased issue with patient compliance, as one size does not fit all. Understanding the different endophenotypes of OSA is essential to achieve the goal of effective therapy and increased compliance and acceptance among patients. Moreover, OSA in each patient can be associated with multiple factors. Studying issues and topics such as craniofacial abnormalities, low arousal threshold, loop gain phenomenon, and other factors affecting OSA can help us understand the pathophysiology and help execute therapeutic decisions. Regarding upper airway pathologies, impaired muscle function and anatomical compromise have been suggested as contributing factors in OSA by causing upper airway obstruction. Understanding the glossal muscles’ anatomical challenges in airway size, such as genioglossus, levator veli palatini, tensor veli palatani, and geniohyoid muscles in affecting hyoid bone position, can be important in the management of OSA [8]. However, surgical correction of upper airway narrowing may not be able to predict the outcomes. For example, work by Wahba and colleagues has shown no improvement in the apnea–hypopnea index (AHI) after lingual tonsillectomy for patients with OSA who have lingual tonsillar hypertrophy [9]. Other non-CPAP devices and interventions can be offered when patients cannot tolerate the first-line treatment. Various pharmacological treatments, such as central nervous system stimulants, antidepressant medications, nasal decongestants, and carbonic anhydrase inhibitors, in managing patients with OSA and upper airway resistance syndrome have been studied to evaluate their effectiveness in decreasing OSA severity and symptoms. The proposed mechanism of action of the pharmaceutical agents, as illustrated in the work of Arredondo et al., helps us improve our understanding of the pharmacological management of OSA–which acts as an alternative to CPAP therapy [8].

Lately, there has been a greater focus on patient-centered therapy. Identifying OSA pathologies and the need of the patients can help in individualizing the therapies. As the prevalence of obesity is increasing globally, the prevalence of OSA is expected to rise. It is time to devote more resources to identifying specific endophenotype abnormalities among individuals and devise individualized therapy, which can help improve the probability of success in compliance and, henceforth, treatment of OSA. Machine learning and AI techniques are making strides in trying to help diagnose OSA in asymptomatic individuals at low or minimum cost. Moreover, nothing is complete without addressing the role of patient-centered treatment for OSA, which can decrease costs by preventing the morbidity and safety risks associated with OSA.
